# Understanding the contextual functions of C1q and LAIR-1 and their applications

**DOI:** 10.1038/s12276-022-00774-4

**Published:** 2022-05-13

**Authors:** Myoungsun Son

**Affiliations:** 1 Institute of Molecular Medicine, The Feinstein Institutes for Medical Research, Manhasset, New York USA; 2 Department of Molecular Medicine, Donald and Barbara Zucker School of Medicine at Hofstra/Northwell, Hempstead, New York USA

**Keywords:** Autoimmunity, Mechanisms of disease

## Abstract

The importance of the complement component C1q has been highlighted by its involvement in autoimmunity, infection, inflammatory diseases, and tumors. The unique tulip-like structure of C1q has both a collagen-like stalk (C1q tail) and heterotrimeric globular head (gC1q), each with different binding specificities, and the binding of these components to their respective receptors leads to functional complexities in the body and bridges innate and adaptive immunity. This review describes the fundamental roles of C1q in various microenvironments and focuses on the importance of the interactions of C1q and its receptors with the inhibitory receptor LAIR-1 in maintaining homeostasis. Current therapeutic opportunities modulating LAIR-1 are also discussed.

## Immunology: complement protein in health and disease

Research into the activities of the protein C1q, involved in a cascade of molecular interactions of the immune response called complement activation, is revealing new details of the protein’s role and opening up possible new therapeutic opportunities. Myoungsun Son at Feinstein Institutes for Medical Research in Manhasset, USA, reviews the involvement of C1q in infection, autoimmunity, inflammatory diseases and tumors. The interaction of C1q with a receptor protein called LAIR-1 seems to be particularly significant. LAIR-1 is present in the membrane of most blood-forming cells and is involved in maintaining the healthy balance of cellular activities referred to as homeostasis. Emerging research suggests that targeting the interactions between C1q and LAIR-1 could enable the development of new treatments for many diseases, including inflammatory diseases, the autoimmune condition lupus, a variety of cancers, and possibly Covid-19.

## Introduction

C1q, an evolutionarily ancient protein canonically known as the initiator of the classical complement pathway, is increasingly appreciated to have a variety of complement-independent functions in innate and adaptive immunity. The structure of C1q, consisting of its globular head (gC1q) and its collagen-like stalk (C1q tail), enables it to interact with multiple binding partners in circulation and on the cell surface to influence local and systemic immune functions. However, current gaps in knowledge include why C1q deficiency predisposes to development of systemic lupus erythematosus (SLE) and how C1q impacts tolerance by suppressing the immune response. This review provides an overview of the current literature on C1q and its receptors. Understanding the downstream consequences of C1q-targeted therapies will be critical for their success in the clinic. Along with these interests, the present review will focus on the role and therapeutic potential of the inhibitory receptor LAIR-1, which plays a significant role in C1q’s maintenance of homeostasis and prevention of autoimmunity.

### Characteristics of C1q

As previously well reviewed^1–4^, the first complement component, C1q, is an ~460 kDa macromolecular glycoprotein that has a tulip-like structure and is found circulating in the blood. C1q is composed of 18 subunits (6 C1qa, 6 C1qb, and 6 C1qc subunits) that contain an N-terminal triple-helical collagen-like region (C1q tail) and a C-terminal globular head region (gC1q)^5^. The C1q tail has the repeating sequence Glycine-X–Y (where X is any amino acid and Y is proline or hydroxyproline), which is also found in collagens^1,6,7^. The transcription factors IRF8 and PU.1 upregulate the synchronized expression of the three chains of C1q^8^. In addition, MafB is one of the critical regulators of C1q production^9^. MafB directly regulates all *C1q* genes, including the promoter regions of *C1qa*, *C1qb*, and *C1qc*. However, the regulation of C1q production at the molecular level is still incompletely understood.

The hematopoietic system is the primary source of C1q production. Although most components of the complement cascade are produced in the liver, C1q is constitutively synthesized by monocytes, macrophages, dendritic cells (DCs), and microglia^10^. Transplantation of wild-type bone marrow to C1q-deficient mice results in a complete restoration of circulating serum levels of C1q^11^. Inappropriate circulating C1q levels are associated with almost all inflammatory or inflammation-related diseases, including cancer, Alzheimer’s disease (AD), and metabolic disease^12^. Toll-like receptor (TLR) ligands induce C1q production by macrophages or DCs in infectious and inflammatory diseases. Moreover, serum C1q levels are also increased with aging but are similar between men and women^13^. Age-related increases in C1q may play an active role in inhibiting muscle repair and regeneration, as evidenced by a study that found that the administration of C1q suppressed muscle regeneration^14^. In the context of tumor biology, C1q is primarily expressed in vascular endothelial and fibroblast cells and in infiltrating monocytes and is associated with tumor invasion^15^. C1q is also highly expressed in the stroma and vascular endothelium in the tumor microenvironment (TME) and acts to enhance tumor progression by promoting angiogenesis^16^.

C1q exhibits diverse binding abilities of cell surface receptors via its gC1q and C1q tail regions. The diversity of C1q functions is related to its domains and differs for each receptor, as its interaction with different receptors will result in different downstream signaling events, which can have inflammatory or anti-inflammatory effects. The interactions of C1q with receptor for advanced glycation end products (RAGE), CD91, scavenger receptor class F member 1 (SCARF-1), CD33 (Siglec-3), integrins and dendritic cell-specific intercellular adhesion molecule-3 grabbing nonintegrin (DC-SIGN), calreticulin and leukocyte-associated immunoglobulin-like receptor 1 (LAIR-1, CD305), and discoidin domain receptor (DDR) 1/2 have been thoroughly and critically reviewed^4,17–20^. Nonetheless, it is important to further assess the consequences of C1q in hematopoietic and nonhematopoietic cells.

### Immune functions of C1q

C1q affects the overall state of the immune system to promote tolerance and quiescence. During the steady state, circulating C1q contributes to the clearance of naturally occurring apoptotic cells; regulates immune cell differentiation by suppressing the differentiation of monocytes into professional antigen-presenting cells, which take part in initiating adaptive immune responses;^21,22^ regulates immune cell polarization, such as by inducing a tolerogenic phenotype in macrophages;^23^ and suppresses proinflammatory cytokine production in innate immune cells, including type 1 interferon (IFN) production by DCs^24^. C1q also upregulates the expression of engulfment machinery, including Mer tyrosine kinase and Gas6, in macrophages^25,26^. C1q enhances macrophage foam cell efferocytosis and cell survival^27^.

**Fig. 1:**
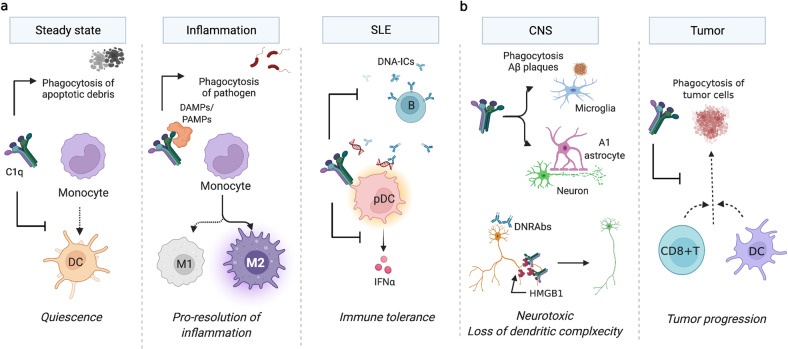
Fundamental roles of C1q. **a** C1q activates the classical complement pathway and induces phagocytosis, allowing apoptotic debris clearance. C1q also maintains quiescence by inhibiting antigen-presenting DC differentiation, which induces adaptive immunity. In the presence of DAMPs or PAMPs, such as HMGB1, C1q not only inhibits proinflammatory cytokine production but also promotes anti-inflammatory (M2) macrophage polarization that is critical for the resolution of inflammation. Once SLE develops, C1q suppresses DNA-containing immune complex (IC)-mediated pDC activation. **b** In contrast, in neuroinflammation or tumors, C1q promotes disease progression. In amyloid β plaques in AD or neuroinflammation, C1q promotes A1 astrocytes and activates microglia that degrade neurons. C1q and HMGB1 promote the loss of dendritic complexity and cognitive impairment. C1q inhibits CD8 + T cells and DCs that kill tumor cells.

In inflammation, C1q acts as an opsonin by binding pathogens, foreign organisms, and apoptotic debris. C1q-dependent engulfment of apoptotic cells is essential for preventing autoimmunity^28^. C1q can interact with various proteins and form protein complexes in particular microenvironments. C1q in human serum binds danger-associated molecular patterns (DAMPs) released from apoptotic and necrotic cells, including phosphatidylserine, nucleic acids, and high mobility group box protein 1 (HMGB1)^3,29–32^. C1q also binds pathogen-associated molecular patterns (PAMPs) in response to infection, including lipopolysaccharides. In the presence of HMGB1, C1q diminishes proinflammatory cytokine production and directs macrophage polarization, leading to the generation of proresolving macrophages (M2-like). Human monocytes cultured with a combination of C1q and HMGB1 upregulated Mer and modulated T-cell proliferation (Fig. 1a). C1q opsonizes oxidized or acetylated lipids and promotes lipoprotein clearance through M2 macrophage polarization in atherosclerosis^33,34^. Moreover, C1q impacts brain inflammation and atherosclerosis^35^. C1q–apolipoprotein-E (ApoE) complexes have emerged as markers for ongoing complement activity in atherosclerosis in vivo. C1q can also bind to advanced glycation end products (AGEs) and facilitate their removal in atherosclerosis^36^.

C1q is of particular interest in systemic lupus erythematosus (SLE), as C1q deficiencies strongly predispose to SLE development^3,11,37^. SLE is a systemic autoimmune disease, but the multifaceted pathogenic mechanisms leading to inflammation and organ damage are not fully understood. However, dysregulation of several functions of C1q is strongly related to several hallmark features of SLE pathology^38–41^. Of particular note are the defective clearance of apoptotic debris^42^ and IFN-α production by DCs induced by SLE autoantibodies and immune complexes^21,43^ (Fig. 1a). In addition to SNPs and other mutations that can alter the expression or function of C1q in some patients, one-third of SLE patients have anti‐C1q antibodies, which can contribute to pathology by inhibiting both C1q opsonization abilities and interaction with inhibitory receptors^44^. Notably, more than 90% of patients have proliferative lupus nephritis^45,46^.

Wound healing integrates various resident and migratory cells, the extracellular matrix, growth factors, and cytokines involved in inflammation, proliferation, and tissue remodeling^47^. C1q promotes the regeneration process and favors wound healing by stimulating angiogenesis in a complement-independent manner^48^. DDR2 is a receptor for C1q that is involved in wound healing and is present on the surface of epithelial cells. C1q and DDR2 binding improved cell migration and induced MMP2 and MMP9 expression in wound healing in vitro^18^.

In the brain, C1q is required for normal neuronal maturation, as it directs microglia to synapses to be eliminated in the process of synaptic pruning^49–51^. C1q expression is upregulated in neuronal injury and early in neurodegenerative disorders such as AD. Indeed, C1q protein levels dramatically increase in the normal aging mouse and human brain^52^. Similar to its functions in the periphery, C1q influences cell polarization in the CNS; astrocytes exist in two reactivity states, A1 and A2, analogous to the M1 and M2 states of macrophages^53^, wherein A1 astrocytes are more inflammatory and A2 astrocytes are generally considered to be neuroprotective. Interestingly, however, increased C1q in the CNS is associated with a more rather than less inflammatory state; microglial secretion of C1q in combination with IL-1 and TNFα induces A1 astrocytes, and mice deficient in C1q have significantly decreased A1 astrocyte reactivity compared to WT controls^53^. Our group has discovered that C1q is a critical component of long-term neuronal damage due to dendritic loss and the cognitive dysfunction associated with this loss in the context of autoantibody-mediated neuronal damage^54^. A subset of SLE-associated autoreactive antibodies (termed DNRAbs) bind double-stranded DNA and cross-react with the excitatory N-methyl-D-aspartate receptors in the brain, causing acute excitotoxicity in the neurons followed by sustained impairments in neuronal integrity and spatial memory. This process is dependent on both microglia and C1q, which colocalizes with synaptic proteins on dendrites, likely tagging the synapses for microglial elimination in a maladaptive form of its normal homeostatic function of synaptic pruning (Fig. 1b). Anti-C1q monoclonal antibodies reduced neuronal damage in AD^55^, and in a tau-induced AD model, microglia-mediated synapse loss was prevented upon administration of a C1q antibody, and synaptic density was recovered. Therapeutics targeting C1q are currently in development, including a nanobody specific for C1q that competitively prevents C1q from binding to IgG and IgM, effectively blocking complement activation by the classical pathway^56^. However, the side effects of prolonged C1q inhibition and whether continued C1q inhibition may result in an increased risk of infection or autoimmunity are unknown^39^.

C1q has protumorigenic properties in the TME^16,57^. Tumors developing in WT mice display early deposition of C1q, higher vascular density, and an increase in the number of lung metastases compared with those developing in C1qa-deficient mice. Moreover, C1q inhibits CD8 + T-cell activation, proliferation, and cytotoxic functions, a situation that may occur in the TME via modulation of the mitochondrial metabolism of CD8 + T cells^58,59^ (Fig. 1b).

C1q may also be detrimental in the context of COVID-19 infection caused by SARS-CoV-2^60^. Clinical studies have shown that defects in type I interferon (IFN) production or antibodies to IFN appear to correlate with severe COVID-19 infection^61^, and anti-IFN antibodies in critical COVID-19 correlate with a poor interferon signature gene response and upregulation of LAIR-1, an inhibitory C1q receptor in PBMCs^62^. The genome of SARS-CoV-2 encodes four major structural proteins: the spike (S) protein, nucleocapsid (N) protein, membrane (M) protein, and envelope (E) protein, all of which are required to produce a structurally complete viral particle^63^. S1, N, M, and E all bind to C1q and activate both the complement pathway and kinin-kallikrein systems^64^. Viral pneumonia has been associated with complement activation and respiratory failure^65^. More studies are needed to show whether C1q and/or LAIR-1 are involved in SARS-CoV-2 infection.

### Leukocyte-associated Ig-like receptor-1 (LAIR-1)

**Fig. 2:**
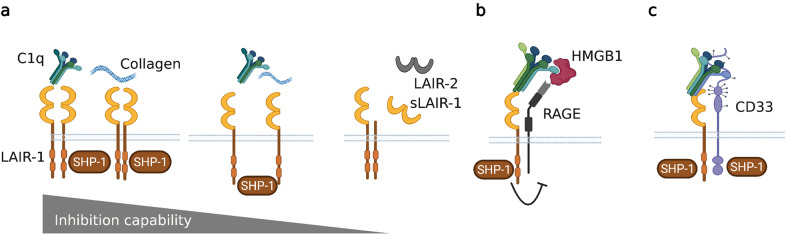
Regulation of LAIR-1-mediated inhibition. **a** The levels of expression of the receptor and soluble forms of LAIR-1 and LAIR-2 alter the strength of LAIR-1 inhibition. In addition, partnering of existing ligands with other receptors, such as (**b**) RAGE or (c) CD33, may also alter the function of LAIR-1.

LAIR-1 is expressed on most hematopoietic cells. LAIR-1 has a large availability of ligands in both circulation and tissues, indicating a need for tight regulation of its interactions^66^. LAIR-1 binds to collagens with high affinity, especially collagen I and III. Additionally, the major LAIR-1-collagen binding site is in the conserved sequence of Gly-Pro-Hyp repeats, present in collagen and C1q^67,68^. LAIR-1 contains two ITIMs that negatively regulate intracellular signaling through SHP-1 binding associated with various phases of the immune response (reviewed by Meyaard^69^). The continuous interaction between C1q or collagens and LAIR-1 may control the inhibition capacity of LAIR-1. LAIR-1 is primarily regulated by its level of expression (Fig. 2a adopted from Meyaard^69^). LAIR-1’s activity is also controlled by secretion of its soluble forms, which include the splice variant LAIR‐2 (CD306) and a shed form of LAIR‐1 (sLAIR‐1), which antagonize LAIR‐1 by acting as decoy receptors^69^. In vivo, LAIR-2 can be found in urine from pregnant women, in fluids such as synovial fluid from rheumatoid arthritis and osteoarthritis patients, and in the circulation of patients with autoimmune thyroid disease^70,71^. Soluble forms of LAIR-1 have been utilized as a therapeutic to reverse immune suppression^72^.

LAIR-1 is highly expressed on monocytes and plasmacytoid DCs. LAIR-1 is also expressed on immune cells in the skin, mainly on tissue-resident CD14 + cells, macrophages and CD11c + DCs^66^. LAIR-1 is consistently upregulated on monocytes and DCs during the inflammatory phase of the immune response and tends to return to normal expression levels during the resolution phase. In tumors, high expression of LAIR-1 has been reported in hematopoietic malignancies and kidney, breast, and ovarian cancers^73^. LAIR-1 is particularly enriched in nonclassical (patrolling) monocytes. LAIR-1-deficient mice have lower Ly6C expression in the steady state lung and enhanced metastatic melanoma in the lung^57^. Overexpression of LAIR-1 was associated with worse overall survival in patients with hepatocellular carcinoma^74^. In contrast, LAIR‐1 expression is lower in pDCs and B cells in SLE patients than in healthy donors, and increased IFN-α and antibody secretion result due to the lack of LAIR-1 inhibition^75,76^.

Activation of LAIR-1 inhibits proinflammatory M1-like macrophage differentiation and promotes alternative activation of macrophages^66,77^. Consequently, LAIR-1 partners with other receptors to improve its own function (Fig. 2b). The C1q-HMGB1 complex polarizes monocytes to anti-inflammatory M2-like macrophages, a pathway mediated through RAGE and LAIR-1 crosslinking, which depends on the relative levels of C1q and HMGB1^32^. CD33 is another inhibitory receptor crosslinked by C1q^20^. C1q specifically binds to CD33 and gC1q, and the C1q tail engages LAIR-1 improve inhibition. The decreased LAIR-1 and CD33 expression on SLE monocytes, along with the frequent abnormalities related to C1q in SLE, suggest that C1q/CD33/LAIR-1 inhibitory networks are disrupted in SLE (Fig. 2c).

Further studies identified that LAIR-1 is an immune inhibitory receptor for collagen domain-containing proteins, including C1q, surfactant protein D (SP-D) and adiponectin^78,79^. C1q belongs to the collectin family, including mannose-binding lectin, SP-A, SP-D, and ficolin, which are pattern recognition proteins^80^. SP‐D also engages LAIR‐1 and inhibits FCαR‐mediated reactive oxygen species production by a human myeloid leukemia cell line^81^. gC1q has a structure similar to that of tumor necrosis factor (TNF) and belongs to the C1q/TNF superfamily (CTRP), which is involved in inflammation^82,83^. Among the CTRP family members, adiponectin behaves similarly to C1q by stimulating Mer tyrosine kinase-dependent engulfment of apoptotic cells^26^. In addition, adiponectin’s globular domain inhibits T-cell activation by interacting with LAIR-1^79^.

In summary, the distribution of collagens, C1q, adiponectin, and SP-D in the body locally regulates the function of LAIR-1 to avoid immune dysfunction. In addition, the interaction of LAIR-1 with C1q (and other ligands) can control immune cells in various stages of the inflammatory response. Thus, LAIR-1 is a targetable receptor that dampens immune reactions.

### LAIR-1 modulators as potential therapeutics

Since the LAIR-1 gene was identified in 1997 by the Meyaard group^84^, studies have demonstrated a critical role for LAIR-1 in the immune imbalance of autoimmune diseases and cancers. Its interactions with C1q alone mediate a major inhibitory pathway for the innate immune response during homeostasis as well as during inflammation and RA and SLE progression^75,85^. Remarkably, current studies suggest that utilizing C1q and collagen or synthetic peptides to modulate LAIR-1 is a beneficial therapeutic strategy at the molecular level in many diseases under appropriate conditions.

LAIR-1 agonistic antibody (anti-LAIR-1) enhances the activity of LAIR-1. In RA, collagen can suppress the T-cell cytokine response through the action of LAIR-1, and treatment with anti-LAIR-1 ameliorated RA severity^85^. In the collagen-induced arthritis (CIA) model and DR-1 transgenic mice, CD3-induced cytokine secretion was significantly suppressed in the presence of collagen, whereas LAIR-1-deficient splenocytes showed no attenuation. Treatment with anti-LAIR-1 significantly attenuated CIA in LAIR-1 wild-type mice. Type II collagen-administered B6.DR1/LAIR-1-deficient mice developed more severe arthritis than wild-type mice^85^.

C1q administration also ameliorates airway inflammation by activating LAIR-1^86^. The findings of Helou et al. suggest that the LAIR-1 pathway is crucial for regulating allergic airway inflammation because it suppresses the activity of type 2 innate lymphoid cells (ILC2s). Crosslinking of LAIR-1 by its known ligand, C1q, decreased type 2 cytokine production by ILC2s in vitro, and IL-33-induced allergic airway inflammation and airway hyperreactivity in humanized mice was significantly reduced^86^.

Similar to C1q, collagen is beneficial for LAIR-1 activation^87^. Some bioactive regions play a role in mediating natural immune cell activation and inflammatory responses by engaging LAIR-1. Human collagen III-derived ligand peptide (LAIR1-LP) targets LAIR-1^88^. LAIR1-LP enhances macrophage uptake through interactions with collagen-domain binding surface receptors and inhibits inflammation through interaction with LAIR-1^89,90^. Moreover, LAIR1-LP inhibits the production of proinflammatory cytokines/chemokines upon T-cell activation^87^. Collagen can stimulate M2 polarization of macrophages in vivo. For instance, implantation of collagen gels into injured muscles of mice resulted in an increased number of M2-like macrophages compared to that seen in control mice^91^. Furthermore, during skin wound healing in mice, it was also demonstrated that collagen injected into wounds led to the M2 polarization of macrophages^92^.

LAIR-1 has the capacity to promote tolerogenic immune responses in the context of DAMP release^31,93^. Our group reported that LAIR-1 can influence macrophage polarization in the presence of HMGB1, which binds the activating receptor RAGE to provoke an inflammatory response but promotes a tolerogenic phenotype when crosslinked by C1q and LAIR-1^31^. In the same study, a fusion protein containing the RAGE-binding fragment of HMGB1 and the LAIR-1-binding fragment of C1q crosslinks the two receptors in the same way as HMGB1 and C1q do. HMGB1 increases leukotriene B4 production in activated monocytes, while HMGB1 plus C1q produces specialized pro-resolving lipid mediators and promotes pro-resolving M2-like macrophage polarization^61^.

The soluble form of LAIR-1 (LAIR-2) blocks the detrimental LAIR-1-mediated inhibition in tumor treatment. Collagen in the tumor microenvironment can affect the ability of T cells to kill cancer cells by regulating the migration of T cells into the tumor^94^. The effects of tumor-associated collagen on immune cells could help explain why a high collagen density in tumors is correlated with a poor prognosis^95^. In mouse models of lung cancer, anti-PD-L1 resistance is associated with enhanced deposition of collagen, as well as fewer and more exhausted tumor-infiltrating CD8 + T cells. Combining anti-PD-1 with blockade of LAIR-1 significantly increases the therapeutic efficacy^96^. Abrogating LAIR-1 immunosuppression through LAIR-2 overexpression or SHP-1 inhibition sensitizes resistant lung tumors to anti-PD-1^96^. Currently, a dimeric LAIR-2 Fc fusion protein, NC410, which both targets the tumor ECM and promotes T-cell function through blockade of LAIR-1-mediated inhibition, is being tested in a trial as cancer immunotherapy^72^. In humanized mouse tumor models, NC410 reduces tumor growth dependent on T cells. Immunohistochemical analysis of human tumors shows that NC410 binds to collagen-rich areas where LAIR-1+ immune cells are localized^72^.

## Concluding remarks

Overall, the current review discusses the biological importance of C1q and its partners, mainly LAIR-1, in immunity and their expected therapeutic effects. Although there are still gaps in our understanding of the functions of C1q in complex microenvironments, targeting LAIR-1 will enable the development of new therapeutic strategies for many diseases, including inflammation, SLE, tumors, and hopefully COVID-19.
